# Visualization of Hot Carrier Dynamics in a Single CsPbBr_3_ Perovskite Microplate Using Femtosecond Kerr-Gated Wide-Field Fluorescence Spectroscopy

**DOI:** 10.3390/nano13192701

**Published:** 2023-10-04

**Authors:** Zhenqiang Huang, Wenjiang Tan, Peipei Ma, Lihe Yan, Jinhai Si, Xun Hou

**Affiliations:** Key Laboratory for Physical Electronics and Devices of the Ministry of Education, Shannxi Key Laboratory of Information Photonic Technique, School of Electronic Science and Engineering, Xi’an Jiaotong University, 28 Xianning Road, Xi’an 710049, China

**Keywords:** lead halide perovskites, hot carrier dynamics, optical Kerr gate, ASE, bandgap renormalization

## Abstract

Lead halide perovskites (LHPs) have excellent semiconductor properties. They have been used in many applications such as solar cells. Recently, the hot carrier dynamics in this type of material have received much attention as they are useful for enhancing the performance of optoelectrical devices fabricated from it. Here, we study the ultrafast hot carrier dynamics of a single CsPbBr_3_ microplate using femtosecond Kerr-gated wide-field fluorescence spectroscopy. The transient photoluminescence spectra have been measured under a variety of excitation fluences. The temporal evolution of bandgap renormalization and the competition between hot carrier cooling and the recovery of the renormalized bandgap are clearly revealed.

## 1. Introduction

Lead halide perovskites (LHPs) have excellent optical properties such as large absorption coefficient, low defect density and high photoluminescence quantum yield [1,2]. Due to these excellent optical properties, they have been used in a variety of applications such as solar cells [3,4], light-emitting diodes [5,6,7], photoelectric detectors [8,9,10] and lasers [11,12]. These applications are also a motivation for fundamental research into the intrinsic photophysics of LHPs. The understanding of the ultrafast carrier behavior in LHPs could be useful for the design of photoelectric devices with improved performance.

Recently, the hot carrier dynamics in this type of material have received considerable attention as they are useful for enhancing the performance of optoelectrical devices fabricated from it. For example, the slow cooling of hot carriers in LHPs has been proven to be conducive to improving the energy conversion efficiency of solar cells by realizing the efficient extraction of hot carriers [13]. Generally, the hot carrier dynamics of LHPs can be affected by many-body interactions, including thermal exciton interaction, bandgap renormalization, band filling and so on [14,15,16,17]. All of these effects may occur simultaneously and compete with each other, which makes it challenging to understand the photophysics of LHPs with many-body effects. The study of hot carrier dynamics using methods of ultrafast spectroscopy is critical to understanding the underlying mechanisms of perovskite-based optoelectronic devices. In fact, many experimental studies have tried to understand the hot carrier dynamics of LHPs by using transient absorption measurements. For example, the slow cooling time of 10 ps for hot carriers due to the phonon bottleneck effect was revealed in CsPbI_3_ using transient absorption spectroscopy [18]. A transient energy reservoir was revealed in 2D (BA)_2_PbI_4_ and the hot carriers in the energy reservoir could spontaneously transfer back to the bright states [19]. The band-filling effect of hot carriers was observed in the transient absorption spectra of CH_3_NH_3_PbI_3_ [20]. However, it is difficult to clearly distinguish some hot carrier dynamics processes in LHPs due to the complexity of the transient absorption signals, which consist of stimulated emission, ground-state bleaching, excited-state absorption and so on. In particular, when multiple processes are present simultaneously, it becomes difficult to accurately characterize the dynamics of each process and the competition between them. Compared to transient absorption spectroscopy, time-resolved photoluminescence (PL) spectroscopy can probe excited-state PL dynamics without interfering with other processes such as ground-state recovery and excited-state absorption, offering a clearer interpretation.

In this work, the ultrafast amplified spontaneous emission (ASE) dynamics of a single CsPbBr_3_ microplate were studied using femtosecond Kerr-gated wide-field fluorescence spectroscopy, which can provide insight for the design and performance optimization of LHP-based lasers and light-emitting diodes. The temporal evolution of the transient PL spectra of a single CsPbBr_3_ microplate under different excitation fluences was observed. Based on our results, the temporal evolution of the bandgap renormalization and the competition between hot carrier cooling and the recovery of the renormalized bandgap were revealed.

## 2. Materials and Methods

**Figure 1:**
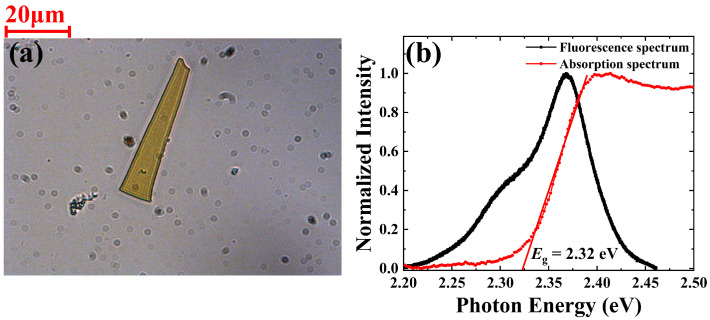
(**a**) Image of the CsPbBr_3_ microplate, (**b**) fluorescence and optical absorption spectrum of the CsPbBr_3_ microplate.

A microplate of single-crystal CsPbBr_3_ served as the sample, and its synthetic method can be found in reference [21]. Figure 1a exhibits an image of the CsPbBr_3_ microplate. Figure 1b contains the optical absorption and fluorescence spectrum. As depicted, the fluorescence spectrum of the CsPbBr_3_ microplate ranges from 2.2 eV to 2.45 eV with a peak photon energy of approximately 2.37 eV. The energy bandgap was determined to be 2.32 eV via linear fitting of the absorption spectrum near the band edge. More material information about the CsPbBr_3_ microplate can be found in the Supplementary Materials (Figure S1). The scanning electron microscope (SEM) and the atomic force microscope (AFM) images of the sample indicate that the sample has a smooth surface and a thickness of 400 nm, respectively (Supplementary Materials, Figure S1a,b). The element analysis results were obtained via energy-dispersive spectroscopy (EDS) (Supplementary Materials, Figure S1c). The calculated element content ratio of the sample is about Cs: Pb: Br ≈ 21: 17: 62, which is consistent with the element ratio of the CsPbBr_3_ perovskite. An optical Kerr gate (OKG) method was employed to measure the time-resolved PL spectra in a single CsPbBr_3_ microplate [22]. A femtosecond laser system was utilized as the light source with a pulse duration of 50 fs and a wavelength of 800 nm at a repetition rate of 1 kHz. The laser pulse was broadened to about 100 fs due to the temporal dispersion introduced by the optical components in the optical path. The 800 nm laser pulses and the 400 nm frequency doubled laser pulses were used as the gating and excitation pulses, respectively. An objective (40×, 0.75 NA, Nikon, Tokyo Metropolitan, Japan) was used to focus the excitation light and collect the fluorescence. To achieve wide-field excitation of the sample, the size of the excitation laser beam size was optimized by adjusting the distance between the sample and the objective lens. CS_2_ filled in a 1mm quartz cuvette was used as the optical Kerr medium. The time resolution of measurement is about 1 ps. The details are described in the supplementary material (Figure S2). All measurements were performed at room temperature.

## 3. Results and Discussion

**Figure 2:**
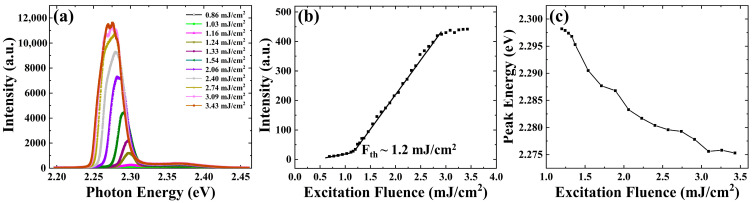
(**a**) Static PL spectra with different excitation fluences, (**b**) dependence of the luminous intensity on the excitation fluence and (**c**) dependence of the ASE peak photon energy on the excitation fluence.

Figure 2a shows the evolution of the PL spectra as the excitation fluence increased from 0.86 mJ/cm^2^ to 3.43 mJ/cm^2^. To analyze the correlation between luminous intensity and the excitation fluence, the integrated spectra intensities at different excitation fluences were calculated and plotted in Figure 2b. It can be seen that the luminous intensity increases slowly as the excitation fluence is increased from 0.86 mJ/cm^2^ to 1.2 mJ/cm^2^. However, the luminous intensity increases faster as the excitation fluence is increased from 1.2 mJ/cm^2^ to 2.74 mJ/cm^2^. This is because the luminescence signal originates from the spontaneous emission process when the excitation fluence is less than 1.2 mJ/cm^2^. In this process, the CsPbBr_3_ microplate spontaneously transits from the high-energy state to the low-energy state and emits photons. As the excitation fluence increases, ASE occurs when the CsPbBr_3_ microplate has been pumped to produce a population inversion but has not reached the lasering threshold. In addition, when the excitation fluence is greater than 2.74 mJ/cm^2^, the luminous intensity increases slowly. Since no laser-induced damage to the material was found, we attributed the slow increase in light intensity to the saturation of light absorption in the CsPbBr_3_ microplate. Because revealing the hot carrier dynamics of materials in the saturated state is also valuable for studying the photophysical mechanisms of materials under such conditions, the dynamics features above the ASE threshold have also been studied in this paper.

Moreover, the balance between optical gain and self-absorption results in a red shift of the ASE peak relative to the fluorescence peak, and the red shift increases with increasing excitation fluence. Figure 2c shows the peak photon energy of the ASE at different excitation fluences. The peak photon energy is less than 2.30 eV, which is smaller than the peak photon energy of 2.37 eV in the fluorescence spectrum shown in Figure 1b. Increasing the excitation fluence from 1.2 mJ/cm^2^ to 3.43 mJ/cm^2^ leads to a red shift of about 23 meV in the peak photon energy of ASE.

**Figure 3:**
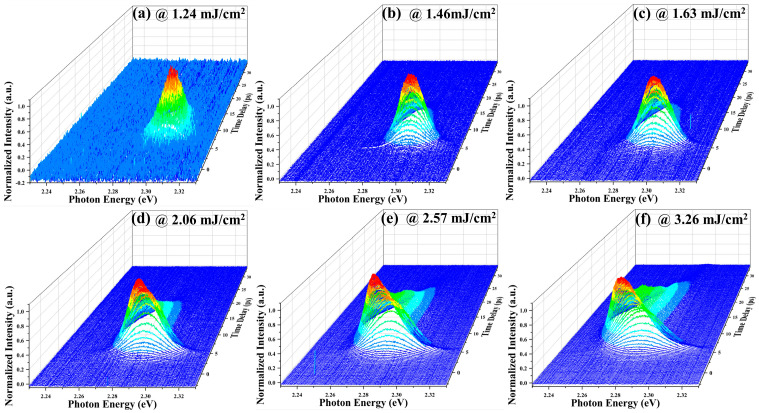
Time-resolved ASE spectra with different excitation fluences: (**a**) 1.24 mJ/cm^2^, (**b**) 1.46 mJ/cm^2^, (**c**) 1.63 mJ/cm^2^, (**d**) 2.06 mJ/cm^2^, (**e**) 2.57 mJ/cm^2^ and (**f**) 3.26 mJ/cm^2^. The results are normalized to the maximum of the luminescence signal.

Furthermore, the time-resolved ASE spectra at different excitation fluences were measured to elucidate the mechanism of the evolution of the ASE spectra under different excitation fluences. The results are shown in Figure 3. It can be seen that the intensities of the transient ASE spectra increase to the maximum with time and then decreases with time at low excitation fluence (Figure 3a). At intermediate excitation fluences (Figure 3b–d), the transient ASE spectra gradually increase with time and a red shift occurs, and then the transient ASE spectra intensity decreases with time with a blue shift. Under the high excitation fluences (Figure 3e,f), the transient ASE spectra gradually increase with time and a larger red shift occurs, and then the intensity of the transient ASE spectra decrease with time with a larger blue shift. 

**Figure 4:**
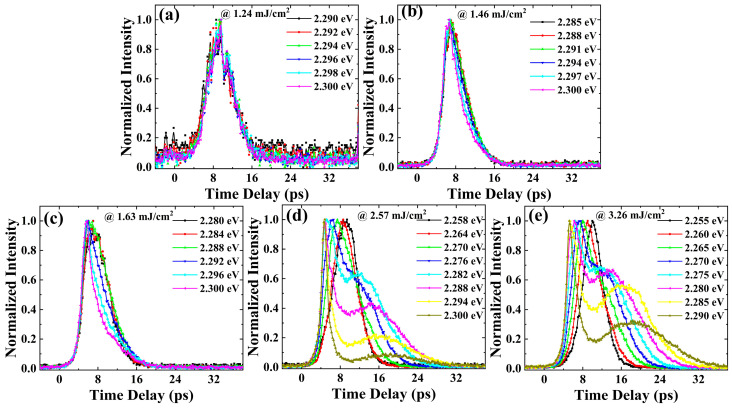
ASE dynamics at different photon energies under several excitation fluences: (**a**) 1.24 mJ/cm^2^, (**b**) 1.46 mJ/cm^2^, (**c**) 1.63 mJ/cm^2^, (**d**) 2.57 mJ/cm^2^ and (**e**) 3.26 mJ/cm^2^.

To analyze the evolution trend of this ASE clearly, we compared the ASE dynamics at different photon energies under several excitation fluences, and the results are shown in Figure 4. The time delay at 0 is defined as the arrival time of the excitation light pulse. It can be seen that the decay processes of different photon energies are basically no difference when the excitation fluence is 1.24 mJ/cm^2^. The transient ASE signal appears with a rise time of about 8 ps and then the intensity of the transient ASE signal attenuates within the delay time of 8 to 16 ps (Figure 4a). This rise time of the transient ASE signal is a consequence of the thermalization and the cooling of the carriers, which is described below. 

Since ASE is a stimulated radiation process, the shift from individual to collective emission leads to accelerated radiation recombination, resulting in a shorter lifetime of ASE than that of the fluorescence processes. As the excitation fluence increases, the decay process at different photon energies shows obvious differences. Though the rise time of the transient ASE signal is basically the same, the intensity of the transient ASE signal attenuates faster at a higher photon energy (Figure 4b,c). When the excitation fluence is greater than 2.57 mJ/cm^2^ (Figure 4d,e), it can be seen that the rise time of the transient ASE spectra at higher photon energies is earlier than that at lower photon energies. For example, when the excitation fluence is 2.57 mJ/cm^2^, the difference in the rise time of the transient ASE signal at the photon energies of 2.3 eV and 2.258 eV is about 4.5 ps. In addition, the transient ASE signal attenuates faster at a higher photon energy, which is similar to the results at intermediate excitation fluence. The intensity of the ASE signal at a high photon energy decreases rapidly with time, but as the time delay increases to around 12 ps, the ASE signal with the high photon energy undergoes a process of re-enhancement and then attenuation. In addition, the transient ASE signal at high photon energies attenuates slower than that at low photon energies after the establishment of the second peak of the transient ASE signal (Figure 4d,e).

**Figure 5:**
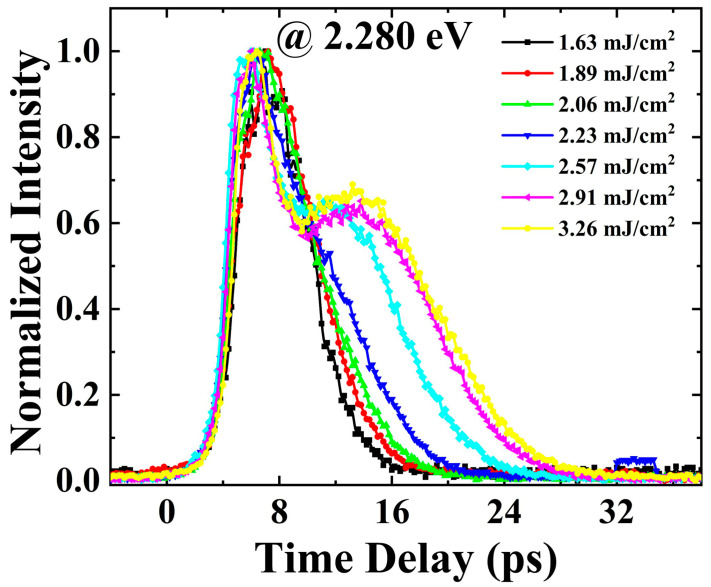
ASE dynamics at the photon energy of 2.28 eV under different excitation fluences.

In order to clearly see the changes in ASE signal intensity with higher photon energies under different excitation fluences, we further compared the ASE dynamics at the photon energy of 2.280 eV under different excitation fluences, and the results are shown in Figure 5. It can be seen that the rise time of the transient ASE signal gradually advances as the excitation fluence increases. After the first peak of the transient ASE signal is established, the transient ASE signal attenuates faster as the excitation fluence increases. With the further increase in the excitation fluence, the transient ASE signal exhibits a process of increasing again and then decaying with time.

**Figure 6:**
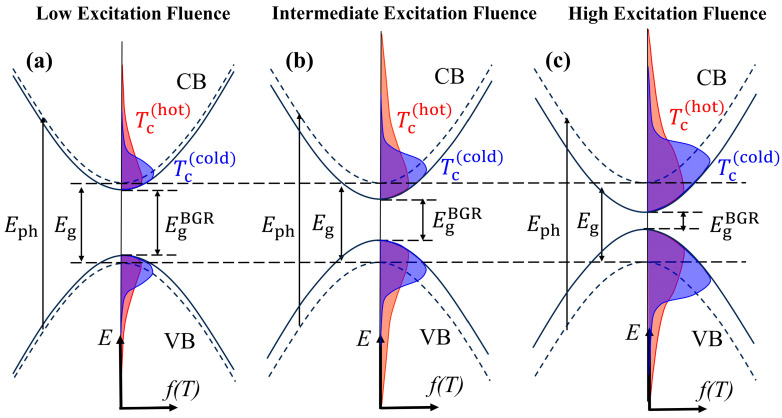
Physical model of hot carrier cooling and bandgap renormalization in different excitation fluences: (**a**) low excitation fluence, (**b**) intermediate excitation fluence and (**c**) high excitation fluence.

In order to explain the above phenomenon, we propose the following physical model (Figure 6). The fundamental mechanism underlying the previously mentioned experimental phenomenon is as follows: When the material is excited by an excitation pulse with a photon energy of *E*_ph_, electrons in the valence band (VB) are excited into the conduction band (CB). The interaction of the photoexcited carriers leads to bandgap renormalization, which moves the bandgap from *E*_g_ to $E_{g}^{\mathrm{BGR}}$, with an established timescale of typically sub-picoseconds. The free carriers complete the thermalization in about 100 fs via carrier–carrier scattering [23]. After rapid thermalization, the carriers remain in a Fermi–Dirac distribution with a high effective carrier temperature ($T_{c}^{\mathrm{hot}})$. Compared to cold carriers with low carrier temperatures ($T_{c}^{\mathrm{cold}})$, hot carriers with high carrier temperatures occupy fewer states at the new, lowered band edge. Subsequently, the process of hot carrier cooling occurs, causing the carriers to be distributed towards a low photon energy, usually within a few picoseconds. Meanwhile, the decrease in hot carrier density, due to radiative recombination luminescence and other carrier depletion processes, leads to the recovery of the renormalized bandgap. 

The degree of bandgap shrinkage caused by the bandgap renormalization depends on the initial hot carrier density. At a low excitation fluence, the bandgap renormalization effect is insignificant (Figure 6a). As the excitation fluence increases, the bandgap shrinkage caused by the bandgap renormalization becomes increasingly significant (Figure 6b,c), which makes the $E_{g}^{\mathrm{BGR}}$ smaller. In addition, with the increase in excitation fluence, the increase in hot carrier density will also cause a more significant phonon bottleneck effect, thereby slowing down the cooling of hot carriers. The cooling of the hot carriers and the recovery of the renormalized bandgap will have a significant effect on the distribution of the hot carriers.

When the excitation fluence is low (Figure 6a), the bandgap renormalization effect is not significant. The main processes that occur here are the cooling of hot carriers and the radiative luminescence of ASE. ASE is generated when the optical gain of some carriers is greater than self-absorption. Since the influence of hot carrier cooling and the recovery of the renormalized bandgap on the distribution of hot carriers are relatively small under low excitation fluence, the ASE signal undergoes normal establishment and attenuation processes, as shown in Figure 4a. When the excitation fluence increases to an intermediate excitation fluence (Figure 6b), the increase in carrier density results in a greater bandgap shrinkage. The phonon bottleneck effect causes the hot carriers to be distributed at a high photon energy, so the density of the carriers at a high photon energy is higher than that at a low photon energy. Due to the high density of hot carriers with high photon energy, stronger carrier–carrier scattering makes hot carriers with high photon energy attenuate faster. Therefore, the intensity of the transient ASE signal attenuates faster at higher photon energies (Figure 4b,c). 

When the excitation fluence increases to high excitation fluence (Figure 6c), the increase in carrier density results in significant bandgap shrinkage. ASE occurs when the material is pumped above the population inversion threshold. At a higher excitation fluence, the spectral range of the ASE is broader (Figure 2a), as the carriers at wider photon energies satisfy the condition for ASE generation. The larger bandgap shrinkage causes the hot carriers to be distributed towards low photon energies under high excitation fluence; this leads to an increase in hot carrier density and population inversion at low photon energies. Therefore, the peak photon energy of ASE appears to have a larger red shift as the excitation fluence increases (Figure 2a). Higher excitation fluence leads to a more significant phonon bottleneck effect, resulting in a higher density of hot carriers distributed at a high photon energy. In addition, the cooling of hot carriers to a low energy level causes the density of carriers at high photon energies to be higher than that at low photon energies. Therefore, the ASE generation conditions are first met at high photon energies and the rise time at high photon energies is earlier than that at low photon energies under high excitation fluences (Figure 4d,e). In addition, significant bandgap shrinkage and hot carrier cooling under high excitation fluences have a significant influence on the carrier distribution at some photon energies. Hot carrier cooling causes hot carriers to be distributed towards a low photon energy, resulting in the red shift of transient ASE spectra. The bandgap shrinkage causes a red shift in the transient ASE spectra. Then, the decrease in hot carrier density, due to radiative recombination luminescence and other carrier depletion processes, leads to the recovery of the renormalized bandgap. The recovery of the renormalized bandgap causes hot carriers to be distributed towards a high photon energy, resulting in the blue shift in the transient ASE spectra. After the carrier cooling is complete, the recovery of the renormalized bandgap will increase the carrier density at some high photon energies, which causes the intensity of ASE signal to increase again (Figure 4d,e). In addition, since the recovery of the renormalized bandgap complements the carriers at high photon energies, the attenuation of hot carriers at high photon energies is slower than the attenuation of hot carriers at low photon energies after the establishment of the second peak of the transient ASE signal (Figure 4d,e).

Since the photon energy of 2.28 eV is relatively high in the ASE spectra, the carrier distribution at this photon energy is mainly affected by the recovery of the renormalized bandgap after the establishment the first peak of the transient ASE signal. Therefore, an increase in carrier density at the photon energy of 2.28 eV during the recovery of the renormalized bandgap results in the generation of a second peak of the transient ASE signal under high excitation fluences (Figure 5). As the excitation fluence increases, the phonon bottleneck effect causes more hot carriers to be distributed at the photon energy of 2.28 eV, which allows the hot carrier at the photon energy of 2.28 eV to reach the generation condition of ASE earlier. Therefore, the rise time of the transient ASE signal gradually advances as the excitation fluence increases (Figure 5).

**Figure 7:**
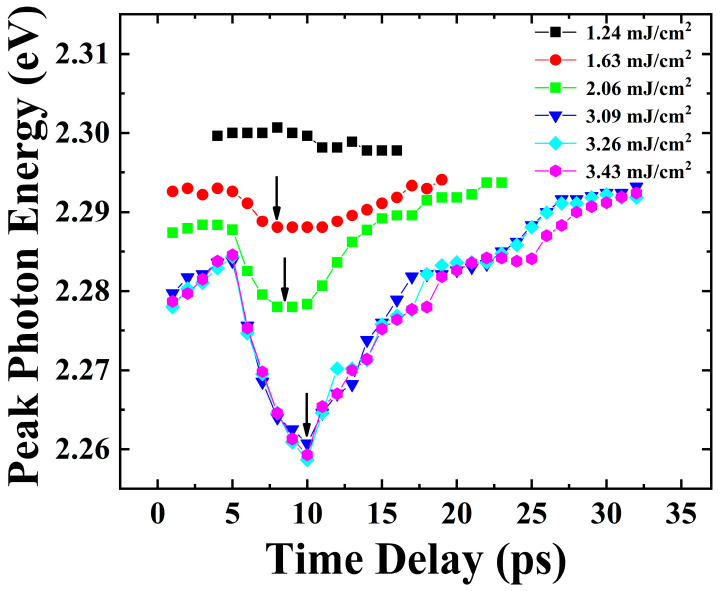
Peak photon energy of transient ASE spectra at different time delays with different excitation fluences.

In order to observe the influence of hot carrier cooling and the recovery of the renormalized bandgap on the distribution of hot carriers at different photon energies, we extracted and plotted the evolution curves of peak photon energy over time under different excitation fluences, and the results are shown in Figure 7. At low excitation fluence (1.24 mJ/cm^2^), it can be seen that there is no change in the peak photon energy. The peak photon energy of 2.3 eV is dependent on the balance between optical gain and self-absorption. Due to the low excitation fluence, the ASE signal is generated in a narrow photon energy range around 2.3 eV, and the influences of the recovery of the renormalized bandgap and hot carrier cooling at the photon energy of 2.3 eV are relatively small, as shown in Figure 6a. Thus, the transient ASE signal at low excitation fluence undergoes normal establishment and attenuation processes.

At intermediate excitation fluences (1.63 mJ/cm^2^ and 2.06 mJ/cm^2^), It can be seen that the peak photon energy of the transient ASE spectra at the initial time delay under higher excitation fluences is lower than that under lower excitation fluences, which is due to the different degrees of the bandgap shrinkage caused by the bandgap renormalization. As shown in Figure 6b, the excitation light with higher excitation fluence will excite higher density of hot carriers, resulting in greater bandgap shrinkage. Therefore, the peak photon energy of the transient PL spectrum at the initial time is lower under a higher excitation fluence due to the bigger bandgap shrinkage. Since the blue shift in luminescence caused by the recovery of the renormalized bandgap is balanced with the red shift in luminescence caused by hot carrier cooling, the initial peak photon energy is still constant within the delay time of 0 to 5 ps under the excitation fluences of 1.63 mJ/cm^2^ and 2.06 mJ/cm^2^. But the increases in the intensity of radiative recombination luminescence cause a decrease in the hot carrier density, and the hot carrier cooling significantly accelerates. The hot carrier cooling process dominates within the delay time of 5 to 8 ps, resulting in the red shift in the transient ASE spectra. After the end of hot carrier cooling, the recovery process of the renormalized bandgap dominates within the delay time of 8 to 25 ps, resulting in the blue shift in the transient ASE spectra. 

At high excitation fluences (3.09 mJ/cm^2^, 3.26 mJ/cm^2^ and 3.43 mJ/cm^2^), it can be seen that in the peak photon energy of the transient ASE spectra a transient blue shift emerges within the delay time of 0 to 5 ps. We speculated that the blue shift is due to the competition between the cooling process of hot carriers and the recovery process of the renormalized bandgap. As shown in Figure 6c, a more significant phonon bottleneck effect under higher excitation fluence makes the cooling of the hot carriers slower, so the recovery of the renormalized bandgap dominates the blue shift in the transient ASE spectra within the delay time of 0 to 5 ps. As the cooling process progresses, the density of hot carriers gradually decreases due to radiative recombination luminescence and other carrier depletion processes, and the cooling process gradually accelerates. Therefore, the cooling process plays a leading role within the delay time of 5 to 10 ps, causing the red shift in the transient ASE spectra. After the end of hot carrier cooling, the recovery process of the renormalized bandgap plays a leading role within the delay time of 10 to 30 ps, resulting in the blue shift in the transient ASE spectra. In addition, we observed that the cooling time of hot carriers increases when the excitation fluence increases, which can be observed by the black arrow in Figure 7. This is because a more significant phonon bottleneck effect slows down the cooling process of hot carriers under higher excitation fluences. 

## 4. Conclusions

In summary, we studied the ultrafast ASE dynamics of a single CsPbBr_3_ microplate using time-resolved microscopic OKG fluorescence spectroscopy. The temporal evolution of bandgap renormalization was revealed under different excitation fluences. The ultrafast ASE dynamics are well explained by hot carrier cooling and bandgap renormalization. When the excitation fluence is low, the influences of hot carrier cooling and the recovery of the renormalized bandgap on the distribution of hot carriers are relatively small, and the ASE signal undergoes normal establishment and attenuation processes. 

When the excitation fluence increases to intermediate excitation fluence, the increase in carrier density results in a greater bandgap shrinkage. The phonon bottleneck effect causes the hot carriers to be distributed at a high photon energy, so the density of carriers at high photon energies is higher than that at low photon energies. Due to the high density of hot carriers with high photon energy, stronger carrier–carrier scattering makes hot carriers with high photon energy attenuate faster. 

When the excitation fluence increases to high excitation fluence, the increase in carrier density results in significant bandgap shrinkage. Higher excitation fluence leads to a more significant phonon bottleneck effect, resulting in a higher density of hot carriers distributed at high photon energies. Hot carrier cooling causes hot carriers to be distributed towards low photon energies, resulting in the red shift in the transient ASE spectra. The bandgap shrinkage causes a red shift in the transient ASE spectra. Then, the decrease in hot carrier density, due to radiative recombination luminescence and other carrier depletion processes, leads to the recovery of the renormalized bandgap. The recovery of the renormalized bandgap causes hot carriers to be distributed towards high photon energies, resulting in the blue shift in the transient ASE spectra. After the hot carrier cooling is complete, the recovery of the renormalized bandgap increases the carrier density at high photon energies, which causes the establishment of the second peak of the transient ASE signal. Finally, a transient blue shift within the delay time of 0 to 5 ps of the peak photon energy of the transient ASE spectra was found, which we attribute to the competition between hot carrier cooling and the recovery of the renormalized bandgap under high excitation fluences.

## Data Availability

The data presented in this study are available on request from the corresponding author.

## Supplementary Materials

The following supporting information can be downloaded at: https://www.mdpi.com/article/10.3390/nano13192701/s1, Figure S1: (a) SEM image of single-crystal CsPbBr_3_ microplate, (b) AFM image of single-crystal CsPbBr_3_ microplate and (c) EDS analysis of single-crystal CsPbBr_3_ microplate.; Figure S2: Experimental setup of the femtosecond microscopic optical Kerr gate system.
