# Supplementary material for: Visualization of Hot Carrier Dynamics in a Single CsPbBr3 Perovskite Microplate Using Femtosecond Kerr-Gated Wide-Field Fluorescence Spectroscopy
Source: Nanomaterials (Basel). 2023 Oct 4;13(19):2701. doi: 10.3390/nano13192701 (PMC10574326; doi:10.3390/nano13192701)
Supplement: Supplementary file 1 [file nanomaterials-13-02701-s001.zip › nanomaterials-2612142-supplementary.pdf]

Supplementary Materials

Visualization of Hot Carrier Dynamics in a Single $CsPbBr_3$ Perovskite Microplate Using Femtosecond Kerr-Gated Wide-Field Fluorescence Spectroscopy

Zhenqiang Huang, Wenjiang Tan *, Peipei Ma, Lihe Yan, Jinhai Si and Xun Hou

Key Laboratory for Physical Electronics and Devices of the Ministry of Education, Shannxi Key Lab of Information Photonic Technique, School of Electronic Science and Engineering, Xi'an Jiaotong University, 28 Xianning Road, Xi'an 710049, China

* Correspondence: tanwenjiang@mail.xjtu.edu.cn

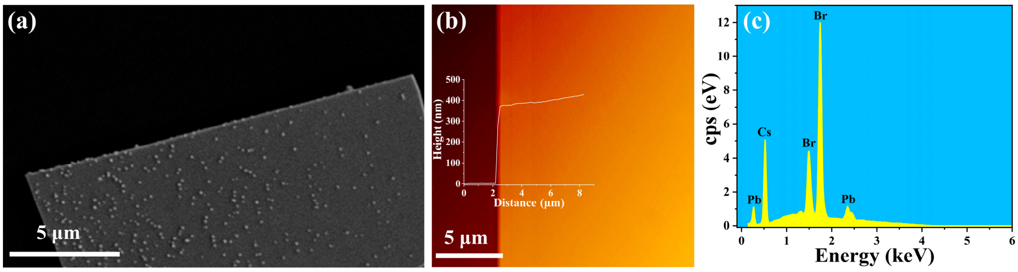

Figure S1. (a) SEM image of single-crystal $CsPbBr_3$ microplate, **(b)** AFM image of single-crystal $CsPbBr_3$ microplate and **(c)** EDS analysis of single-crystal $CsPbBr_3$ microplate.

The experimental setup is shown in Figure S2. The light source is a femtosecond laser system with a pulse duration of 50 fs and a wavelength of 800 nm at a repetition rate of 1 kHz. The laser was divided into two beams in a ratio of 5:5 using a beam splitter (BS). The transmitted part and the reflective part were used as the signal beam and gate beam, respectively.

For the gate beam, a half-wave plate (HWP) was used to change the polarization of the gate pulse. The gate beam first passed through an optical delay line (ODL) and was then introduced to the optical Kerr medium (OKM). The gate beam was used to open the optical Kerr gate (OKG), which consists of a pair of crossed Glan–Taylor polarizers P1 and P2 (PGT5012, Union Optic, Inc., China) and an OKM between them. CS_2 filled in a 1mm quartz cuvette was used as the OKM. The polarization directions of polarizers P1 and P2 were horizontal and vertical, respectively. In order to obtain the maximum transmission efficiency, the polarization of the gate beam was rotated by $\pi/4$ with respect to the polarizer P1 using the HWP. The extinction ratio of the polarizers was greater than 10^5. The angle between the propagation direction of the gate pulse and the signal pulse is about 9°.

The signal beam was doubled in a 1 mm BBO crystal and used to excite the sample, and the residual fundamental laser after doubling in the BBO crystal was filtered using a band-pass filter (BPF, 400-600nm). A long-pass filter (LPF1, >425nm) was used to reflect the exiting light and excite the sample after it was focused through an objective lens (40×, 0.75 NA). The fluorescence was collected by the 40× objective lens and passed through the LPF1. To achieve wide-field excitation of the sample, the size of the excitation laser beam size was optimized by adjusting the distance between the sample and the objective lens. A turn-back mirror M6 was used to switch the signal's optical paths. A charge-coupled device camera (BFLY-U3-13S2C-CS) was used to record the images. The fluorescence was focused into the OKM using lens L1 (focal length, f1 = 75 mm) after being passed through polarizer P1 and collected by lens L2 (focal length, f2 = 100 mm). The gated fluorescence after passing through polarizer P2 was focused and transferred to the entrance slit of spectrograph (Princeton Instruments, SpectraPro - MS3504i) using lens L4 (focus length, f4 =

150 mm). Two adjustable neutral optical attenuators (A1 and A2) were used to adjust the intensity of the gate pulse and excitation pulse. These cutoff filters determine the detection spectral range of the present setup (425–750 nm). The dispersed fluorescence spectrum was recorded using a TE-cooled (−60 °C) CCD camera (Andor, DU970P-UVB).

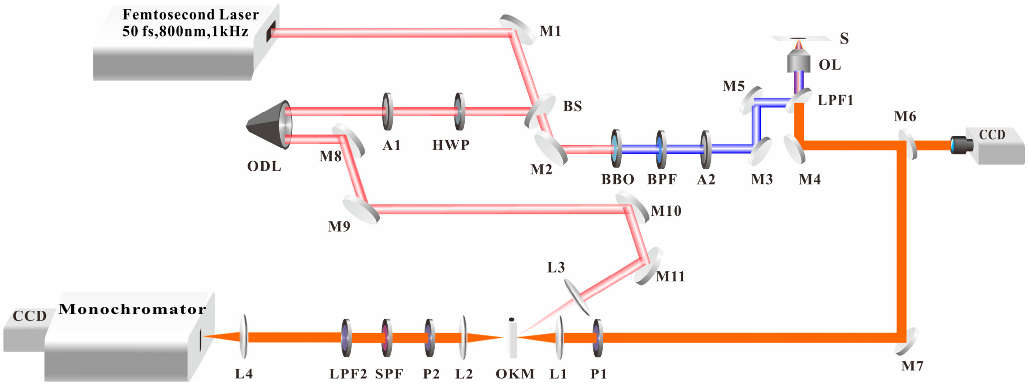

Figure S2. Experimental setup of the femtosecond microscopic optical Kerr gate system. M1-M11: mirrors; L1-L4: lenses; A1 and A2: attenuators; BS: beam splitter; P1 and P2: polarizers; LPF1 and LPF2: long-pass filter; OL: objective lens; BPF: band-pass filter; SPF: short-pass filter; ODL: optical delay line; OKM: optical Kerr medium; HWP: half-wave plate.
